# Survival study of 86 bitches with mammary tumors treated at the Veterinary Hospital of the University Anhembi Morumbi, São Paulo, Brazil

**DOI:** 10.1186/1753-6561-7-S2-P1

**Published:** 2013-04-04

**Authors:** Manoela P Scapinelli, Adriana T Nishiya

**Affiliations:** 1 Veterinary Hospital of Anhembi Morumbi University, São Paulo, SP, Brazil

## Background

Mammary tumors are the most common cancers in dogs. The aim of this study is to analyze the association between the survival rates and the variables: size of tumors, presence of metastasis, type of treatment and stage of malignancy.

## Patients and methods

In this retrospective study, clinical records of 86 animals with 154 mammary formations, treated in 2010-2011 at the Veterinary Hospital of the University Anhembi Morumbi, were analyzed for breed, age, histopathogical type, size, presence or absence of metastases, survival and staging of tumor. Kaplan-Meier log rank analysis was used to evaluate survival.

## Results

Among the 154 tumors analyzed, 149 (96.7%) were neoplasms (benign and malignant) and 5 (3.3%) were non-neoplastic lesions. For statistical analysis, mongrel (30.2%) and Poodles (26.7%) were a lot more affected breeds, age between 9 and 12 years (62.8%) was the most frequent of all, 60,4% (90/149) of neoplastic formations were benign and 39,6% (59/149) were malignant. 24,4% (12/49) of all malignant tumors patients had metastasis. Dogs whose tumors were less than 4 cm had no significantly increased duration of survival with a median of 15,3 month versus 17,4 months for dogs with tumors greater than 4 centimeters (p=0,327). Dogs with metastasis lived a median of 10,7 months versus 18,3 months for dogs without them (p<0,01). Patients treated with surgery had 17,3 months of median survival rate versus surgery plus chemotherapy submitted dogs had 13,8 months (p=0,319). Dogs with malignant tumors in stage I, II, III, IV and V had, respectively, 21, 18,4 , 14,9 , 17,2 and 8,8 months of survival (p<0,01).

## Conclusions

Survival analysis of patients with malignant mammary tumors indicated that size did not influence survival time, the animals live less if had metastasis, the type of treatment (only surgery or surgery plus chemotherapy) did not change survival rate and there was a tendency of decrease survival rate for animals in more advanced clinical staging.

